# Investment into the future of microbial resources: culture collection funding models and BRC business plans for biological resource centres

**DOI:** 10.1186/2193-1801-3-81

**Published:** 2014-02-12

**Authors:** David Smith, Kevin McCluskey, Erko Stackebrandt

**Affiliations:** CABI, Egham, Surrey, TW20 9TY UK; University of Missouri, Kansas City, USA; MIRRI, c/o DSMZ, Braunschweig, Germany

**Keywords:** Microbial resource centres, Biological resource centres, Global biological resource centres, Bioeconomy, Networks, MIRRI

## Abstract

Through their long history of public service, diverse microbial Biological Resource Centres (mBRCs) have made myriad contributions to society and science. They have enabled the maintenance of specimens isolated before antibiotics, made available strains showing the development and change of pathogenicity toward animals, humans and plants, and have maintained and provided reference strains to ensure quality and reproducibility of science. However, this has not been achieved without considerable financial commitment. Different collections have unique histories and their support is often tied to their origins. However many collections have grown to serve large constituencies and need to develop novel funding mechanisms. Moreover, several international initiatives have described mBRCs as a factor in economic development and have led to the increased professionalism among mBRCs.

## Introduction

The provision of microbial resources for research is now recognised as being an essential component in the advancement of the life sciences. The Organisation for Economic Cooperation and Development (OECD) established the concept of Biological Resource Centre (BRC) and stressed their importance in the developing bioeconomy. The general concept of a BRC presented at that time includes service providers and repositories of the living cells, genomes of organisms, and information relating to heredity and the functions of biological systems. BRCs contain collections of culturable organisms (i.e. of the four domains of life: micro-organisms, plant, animal, and human cells), replicable parts of these (e.g. genomes, plasmids, viruses, cDNAs), viable but not yet culturable organisms cells and tissues, as well as data bases containing molecular, physiological and structural information relevant to these collections and related bioinformatics (OECD 2007). At the conclusion of the OECD BRC task force work it was considered advisable to coordinate development in the four domains separately because the organisms were handled very differently and because existing collections were embedded in the different communities they serve. Consequently, the microbial community followed up this work with the Global Biological Resource Centre Network demonstration project (Fritze et al. 2012) which focussed on collections of living microorganisms. European member states in the European Strategy Forum for Research Infrastructures (ESFRI) accepted the pan-European research infrastructure Microbial Resources Research Infrastructure (MIRRI) on to their road map for 2010 and its preparatory phase began in late 2012. At a global level, regional initiatives in the USA, Asia, and South America are underway (Smith 2012). The hypothesis is that the full potential of microbial diversity is yet to be harnessed and a coordinated approach to resource provision will accelerate innovation and discovery. This paper discusses the further development and investment needed in the microbial domain and includes the transition necessary from the traditional culture collection, holders of laboratory based living material, to the next generation microbial resource centres.

## Review

### Background

The first public service collection was established by Professor Frantisek Král in 1890 at the German University of Prague (Uruburu 2003) and some of the strains first deposited remain available through the public service collections of today. Shortly after, the collection of the Institut Pasteur was created by Dr. Binot in 1891 (see http://www.pasteur.fr/ip/easysite/pasteur/en/research/collections/crbip/general-informations-concerning-the-collections/iv-the-open-collections/iv-i-collection-of-institut-pasteur-cip). Some of the earliest fungal mBRCs include the Mycothèque de l’Université Catholique de Louvain (MUCL, Belgium) founded in 1892 which currently holds over 25,000 strains of filamentous and yeast-like fungi, representing over 3,300 species of Ascomycetes, Basidiomycetes, Hyphomycetes and Zygomycetes; The Centraalbureau voor Schimmelcultures (CBS) Fungal Biodiversity Centre, founded in 1904 as an institute of the Royal Netherlands Academy of Arts and Sciences, maintains a world-renowned collection of living filamentous fungi, yeasts and bacteria. Other collections have grown by consolidation over many years and the CABI culture collection is an example of this path. While CABI was founded in 1910, the CABI culture collection has its roots in the Imperial Bureau of Entomology which was established in 1913. This was followed in 1920 by the Imperial Bureau of Mycology whose dual foci were the identification of fungal diseases of plants, animals and humans, and the abstracting of the mycological literature. Requiring a collection of reference strains, the collection at the institute formerly known as the Imperial Mycological Institute (IMI), Commonwealth Mycological Institute (CMI), International Mycological Institute (IMI) and now simply CABI, was established in 1947 and now holds approximately 30 000 strains.

Over recent years collections have adopted new technologies to characterise and add value to their holdings. The Fungal Genetic Stock Center (FGSC) at the University of Missouri-Kansas City demonstrates this (McCluskey et al. 2010). The collection, originally founded in 1960 to preserve mutant strains of *Neurospora* and *Aspergillus* currently holds over 19,000 *Neurospora* strains including more than 12,000 *Neurospora* gene deletion mutants, over 2,000 *Aspergillus* strains, and various representatives of other fungal taxa including mating type testers, vegetative compatibility testers, and strains subject to whole genome sequence analysis. Additionally, as molecular genetic technology developed in the 1980’s and 1990’s, the FGSC added cloned genes, cloning vectors, and gene libraries to its holdings. In 2003 and 2004, FGSC accepted nearly 50,000 *Magnaporthe* GMO mutants; in 2005 it began to distribute arrayed sets of knock-out mutants of *Cryptococcus* and *Candida* mutants. The rapid growth holdings at the FGSC has been associated with growth of strain distribution of over 100 fold (1,000 to >100,000 total strains per year).

As the value of microbial germplasm became better understood (Stern 2004), over 600 service collections have been established. The register of culture collections at the World Data Centre for Microorganisms (WDCM) presents data on almost 650 culture collections world-wide holding over 2 million strains of microorganisms, mainly bacteria and fungi. Almost 130 of these are affiliated collections of the World Federation for Culture Collections (WFCC) and as such they have agreed to operate to WFCC guidelines (Anon 2010).

The modern day culture collection has had to adapt to new demands and regulatory requirements and thus the microbial domain Biological Resource Centre (mBRC) has evolved. mBRC status is available to those microbiological resource collections that implement OECD best practice, network their activities nationally and internationally for efficiency and effectiveness, meet mandatory guidance laid down in membership rules, and carry out research to add value to holdings. Ultimately however, it is nations that assign formal mBRC status and while there are many self-declared mBRCs there is no formal mechanism to recognize this status. Many collections meet most of the criteria of a formal mBRC, although many are missing the collaborative, or networking elements, the advanced quality control, and the adherence to international standards and regulations.

This notwithstanding, there is gathering evidence that mBRCs are contributing to discovery and helping provide solutions to societal and economic challenges. The European Consortium for Microbial Resource Centres (EMbaRC) project (http://www.embarc.eu) drew together examples of microbial resource collection success stories (Smith and Day 2012) that demonstrate the value of providing products and services directly to bioindustry to help develop marketable products. The solutions described cover biocontrol agents to control locusts, phages to control human disease, microbial enzyme activity to decontaminate waste water, improved flavour in alcoholic drinks and improvements in food contamination detection and prevention. Such examples of collection to use reveals how microbial resource collections are playing a role in addressing the world’s grand challenges by improving our environment, facilitating food security, and providing products for healthcare. Similarly, the impact of materials has been evaluated and some measures demonstrate that impact is increased more than two fold when materials are publically available (Furman and Stern 2011). The potential is enormous if such activities could be enhanced, multiplied and coordinated through closer relationships between collections and researchers and facilitation of partnerships. The Microbial Resource Research Infrastructure (MIRRI) goals focus European collections on providing high quality resources to support research and development and ultimately this aim (http://www.mirri.org).

The G8 Science Ministers Statement https://www.gov.uk/government/publications/g8-science-ministers-statement-london-12-june-2013 made on 12th June 2013 highlighted the need to improve transparency, consistency and coordination of the global scientific research enterprise to address global challenges. They focussed on antimicrobial drug resistance as a major health security challenge and stressed that research infrastructures are key elements in research and innovation policies. MIRRI intends to address these challenges by working with multidisciplinary partners delivering to them the resources, tools, and services needed to facilitate the discovery of solutions. The microbes are the great pioneers of our planet surviving the extremes and having interesting chemistry to provide answers. Examining organisms from before the introduction of antibiotics and comparing them with resistant strains provides the obvious route to improving our understanding and increasing the opportunity to discover new therapeutic modalities. Understanding the chemistry and seeking those organisms with the properties needed, be they taxonomic relatives or organisms from particular ecosystems, the coordinated efforts of Microbial Resource Centres can accelerate the process. Well described microbial resources will play a key role in underpinning the bio-economy and driving economic growth. To do this there is the need to better utilise microbiological diversity in biotechnology. This is fundamental to the delivery of the bio-economy and to accelerate the discovery of natural solutions to today’s global challenges.

The United States Culture Collection Network (USCCN) is a Research Coordination Network (RCN) for *ex situ* Microbial Germplasm Collections sponsored by the US National Science Foundation (Grant DBI-1203112). The US Culture Collection Network (http://www.usccn.org) brings together scientists working with laboratory based collections of microbes. The network is holding workshops to teach best practices for managing, preserving, and distributing bacteria, fungi, and other microscopic organisms in the context of formal culture collections. Biosecurity and regulatory issues are to be emphasized at workshops. Additional goals include re-establishing a professional society of culture collection researchers in the US, developing internet based collection management tools, and fostering communication between US collections, foreign collections, and international collection networks. The overall aim is to ensure that microbial resources are made available to the scientific community and the culture collections providing them are supported and sustainable. These are common goals shared globally and it is important that lessons learned are shared.

### Funding systems of mBRCs

The relatively higher requirements associated with mBRC status come with additional associated costs that mBRCs must bear. However, the increasing professionalism of BRCs of every type comes at a time when science spending is held constant or is actually reducing. The strategy must therefore be to find cost effective ways to balance operational costs with income and to improve services and outputs to attract investment into mBRCs. Firstly, we need to understand these costs better.

While there is not one model for the operational and financial sustainability of mBRCs, we can learn a lot from the experience of existing culture collections. Recent studies by the OECD and the EMbaRC project have summarised working models of mBRCs (Smith et al. 2013). Although culture collections or mBRCs have similar activities and objectives as repositories and suppliers of living laboratory based biological materials they can be quite different in size, scope and function. Collections can be based around an individual researcher or research team on the other hand they can be large public service collections with a multitude of structures in between. To be an affiliate collection of the WFCC, however, a collection should not be dependent on one individual, but should have some long term institutional commitment. A typical culture collection financial plan involves revenues from fees for products or services, research, or service contracts, but most rely on some form of Governmental or host institutional funding. Additionally, the mBRC or its host may have opportunities for other types of cost recovery activities and these often revolve around expertise and facilities available. The degree to which such activities may actually provide support sufficient to ensure financial sustainability of an mBRC is unproven. Other kinds of funding sources include support from industry, grants from agencies that support research, development of databases and other tools that compliment the core role of mBRCs, and even funding from charitable sources, especially those associated with public health or sustainable development. Evaluating different funding mechanisms through controlled trials has been proposed and this proposal specifically contrasted the scientific method with current expert-panel based evaluation of funding mechanisms (Azoulay 2012). Because this requires both coordination and a sufficient data set for comparison, it is difficult to envision a statistical comparison of mBRC funding mechanisms in the current funding milieu.

#### Costs of running an mBRC

The Organisation for Economic Co-operation and Development (OECD) defined a Biological Resource Centre (BRC) as the next generation of culture collection and genetic resource banks. As such they have both basic costs and the elevated associated costs in running the quality management aspects and improved services and research (Table 1).

**Table 1:** **Collection development**

***Status of collection***	***Characteristics of collection status***
**Basic level**	• Basic methods for Biological material preservation are available
	• Basic documentation level
**Intermediate level**	• Sound facilities
	• Good human resources
	• Good technologies in place
	• Electronic catalogues and data management
	• Operating to international criteria
	• Wide stakeholders’ involvement
	• Involvement into regional/national networking
**High level**	• Sustainable human resource training in relevant to BRC domains
	• Collection quality management is in place
	• Intellectual Property Rights regulations, MTAs; biosafety and biosecurity standards are in place
	• Accredited or certified to the operational and quality levels of the International Standards Organization or
	• Equivalent
	• Clear management program and collection’s strategy in place
	• Sustainable fundraising mechanisms with governmental support
	• Raise of public awareness in the domain of Biological Resources preservation
	• Regular monitoring and adjustment of collection needs
	• Leading activity in the regional/national/international networking
**BRC**	• High quality standards collections accredited and certified to the OECD standards
	• Functioning according to the OECD instruments

All collections need to address three key functions, the ABC of BRCs, Authentication of strains, Best practice in preservation and supply, and Confirmation of validity of the associated information provided. However, the costs associated with these basic tasks can vary, making it extremely difficult to get a meaningful estimate of the average costs of running a mBRC. In the microbial domain, operational costs very much depend on the type and number of strains being preserved, maintained and distributed, the extent of the added value the mBRC provides, and the number and scope of associated services being provided. Regional factors such as the cost of living and salary expectations also impact budget requirements. The cost of accessioning a strain in a World Federation for Culture Collections (WFCC) affiliated collection was shown to vary enormously (Smith 2012); the most expensive quoted was over €3000 but the average cost is over €350. These costs depend on the difficulties in handling the organisms, for example the investment in time in isolating single cells to remove contamination, and defining optimum growth conditions. Costs also depend upon the techniques utilized to preserve the different organisms and the extent to which they are characterized, for example some collections include sequencing costs or other methodologies such as metabolic or protein profiling for checking authenticity (Smith 2012; Smith *et al*. 2013) or molecular tools such as Ribotyping (Kostman et al. 1995) or MALDI-TOF (Matrix Assisted Laser Desorption/Ionization, Time of Flight) mass spectroscopy (Lay 2000). Once strains are accessed into the collection they must be stored indefinitely and costs vary depending upon the methodology used and the validated life span of the specimen. While it may cost only a few cents to store an ampoule in liquid nitrogen for a year, a collection of 1,000 strains may expect to spend €1.500-3,750 per year for liquid nitrogen. Larger collections, which are able to secure lower costs, may incur liquid nitrogen costs around €15,000 to store a collection of 20,000-30,000 strains. Freeze dried ampoules are more costly to generate and should be stored at refrigerated temperatures, but their cost of storage would be less. Distribution of strains depends on the nature of the strain and the format distributed. Some strains are simply packaged in UN approved packaging materials and sent by courier other cells may need to be shipped cold or even frozen on dry ice. Similarly, some collections send lyophilized ampoules while other collections re-activate strains and send living cultures.

Staffing a collection is by far the largest cost associated with running an mBRC. A collection of 5,000 strains growing at 500 strains per year and supplying 2,000 strains per year should require at least three members of staff to cope with the authentication, preservation and distribution. An additional staff member would be required to cope with implementing quality standards and adherence to regulations. Moreover, the taxonomic depth of the collection, or the diversity of these 5,000 strains plays a role in how many staff are required. If all these strains are metabolically very similar, fewer staff is needed than if the strains cover a wide range of physiological diversity, in which case more expertise is required to maintain them. If an identification service is to be provided then this will have additional staffing needs and associated costs. Add to this a collection manager/business developer and researchers, the costs rise quickly. Thus the cost to run a collection with minimal services and research capacity (the latter to improve service to users) may be over €500,000 per year in Europe (Table 2) but will vary depending upon the national and local financial conditions.

**Table 2:** **Collections’ costs**

Cost item	Detail	Cost per year (thousand €)
Staff	3-10 staff (number depends upon the phylogenetic depth, breadth, and related activities)	185-300
Acquisition/deposit of strains	500/year at US$500ea	185
Maintenance of strains	5000 strains	30
Distribution of strains	2000 strains	30
Data management	Proprietary data base	1
Accommodation, utilities etc.	Laboratories etc.	90
Consumables	Ampoules, chemicals,	20
**Total**		**540-730**

This cost varies enormously as examples given below show. CABI Bioservices, which is equivalent to the CABI mBRC, has running costs of almost €1.1 million per annum; it holds almost 30,000 strains of fungi and bacteria, it authenticates all its strains using DNA sequencing (generally the ITS region for fungi, although to differentiate some genera specific genes are sequenced; for bacteria the 16S rRNA). CABI identifies over 2,000 strains for outside users. It has a reduced capacity for acquisition of deposits and supplies only a few hundred strains per year; culture supply amounts to less than 3% of its revenue. The bulk of its revenue comes from the identification service, industrial consultancy services and several contract projects (see below). It has 19 staff and its day to day operations run on a cost positive basis. However, the maintenance of the 30,000 strain archive is partially supported by the CABI member countries and its host institution and these subsidies cover about 10% of costs.

The situation at the Leibniz-Institut DSMZ–Deutsche Sammlung von Mikroorganismen und Zellkulturen GmbH (German Collection of Microorganisms and Cell Cultures) is not typical of the majority of public collections in Europe. DSMZ embraces four different kinds of holdings, i.e. prokaryotes (Archaea and Bacteria), human and animal cell lines, plant virus and plant cell cultures within a single institute with running costs of about €5.6 million per annum (2011) and a revenue from identification (molecular, chemotaxonomic and physiological identification, mycoplasma elimination of cell cultures) and sales of resources of €3.85 million per annum (2011) for these departments. The DSMZ funding agencies (mainly the ministry for Science and Technology) permits the DSMZ to reinvest the majority of this income. The section of prokaryotes, yeast and fungi maintains about 26,000 strains which are curated by 8 scientists and 18 support staff, with annual running costs of about €2.98 million. About 20,000 microbial strains were shipped 2011 to national (40%) and international (60%) customers. The large number of scientific and technical staff is explained by the physiologically and phylogenetically highly diverse nature of resources which span almost all phyla, and the majority of genera and species of prokaryotes. This is exemplified by the maintenance of about 1,500 different growth media. Other microbial collections with less diversity are able to handle large holdings with much fewer numbers of support personnel.

Conversely, even the lower level of the average cost of running an mBRC (€542 K) is very high compared to the budget of the Fungal Genetic Stock Center (FGSC), in Kansas City, Missouri. As is the case with many mBRCs, they do not have support for some of the essential functions at the High level described in Table 2, and only marginally meet the criteria for the intermediate level. For example, they operate with 2.5 full time people comprising of 1 full time manager and 1.5 practical curators. In addition, the director of the FGSC is a full-time faculty member with no day-to-day responsibilities at the collection. The FGSC financial model is one based on national funding and is dictated by the allocated budget which often fluctuates and the trend, like most parts of the world, is down. Recently, the funder has changed their expectations and FGSC can apply for support for up to $165 K/year (€123 K), including overhead which translates to about $100 K (€75 K) for salaries and travel. Clearly this is not enough to sustain the collection and the FGSC and other NSF supported collections are encouraged to identify additional sources of financial support including increased user fees. An additional consideration in these cost comparisons is how the overhead is calculated. For FGSC the overhead is 50% of the nominal amount of the grant–if they get $100 K direct costs, the university requires an additional $50 K in overhead. This is expected to include things like lab and office space, heat/cooling, power, water, janitorial services, administration and so on. Some universities and institutions have much higher overhead, up to 100-150%.

The diversification of activities in the transition from the ‘Culture Collection’ to a microbial domain BRC, or mBRC, anticipates additional costs to support expansion of quality management and regulatory compliance. Quality management and quality control are the basis to create a certified/accredited BRC. To implement these control systems the mBRC must follow the OECD general requirements defined in *OECD Best Practice Guidelines for Biological Resource Centres* (OECD 2007) combined with their national legislation, regulations and policies (e.g. sound facilities, human resources necessary to operate, preservation technologies in place; e-catalogue and data management, operating following national and international criteria including Intellectual Property Rights (IPR) regulations, material transfer agreements to formalise exchange of materials, biosafety and biosecurity standards, etc.). There have been over 20 collections (Table 3) that have become certified or fully/partially accredited and some of the partners in the Global Biological Resource Centre Network (GBRCN) demonstration project have indicated these costs in their particular situation (Fritze et al. 2012). MIRRI are taking such costs into consideration in the development of their business plan and estimate average costs to be in the order of €60 K for culture collections to become certified or accredited; although some small collections have been able to put in place ISO 9001 series certification for a cost in the order of €18 K. Few collections, or parts thereof, have become accredited; usually standards such as ISO 17025:2005 are limited to certain procedures or services rather than applied to all operations.

**Table 3:** **Accreditated mBRCs**

Acronym	Name	Country	Standard
ATCC	American type culture collection	USA	ISO9001:2008; ISO 17025:2005; ISO Guide 34:2009
BIOCEN (BioCC)	Centro Nacional de Biopreparados	Cuba	ISO 9001:2000
CABI	CABI Genetic Resource Collection	UK	Part ISO17025
CCCM	Czech Culture Collection of Microorganisms	Czech Republic	ISO 9001:2008
CBS	Centraalbureau voor Schimmelcultures	Netherlands	ISO9001:2008
CCOS	Culture Collection of Switzerland	Switzerland	ISO 9001:2008
CCRC	Culture Collection and Research Center, FIRDI	Taiwan	ISO 9001:2000; ISO/IEC 17025:2005
CECT	Coleccion Espanola de Cultivos Tipo	Spain	ISO9001:2008
CRBIP	Collection de l’Institut Pasteur	France	ISO9001:2008
DSMZ	Leibniz-Institut DSMZ-Deutsche Sammlung von Mikroorganismen und Zellkulturen GmbH	Germany	ISO9001:2008
ICLC	Interlab Cell Line Collection	Italy	GMP
IFM	Quality Services Pty Ltd	Australia	ISO/IEC 17025; ISO/IEC17043
IHEM	Institute of Hygiene and Epidemiology, Mycology	Belgium	ISO 9001:2008; ISO/IEC 17025:2005
LMBP	Department of Biomedical Molecular Biology Ghent University Plasmid collection	Belgium	ISO9001:2008
LMG	University of Gent	Belgium	ISO9001:2008
MUCL	Mycology, University Louvain la Neuve	Belgium	ISO9001:2008
MUM	Micoteca da Universidade do Minho	Portugal	ISO9001:2008
NBRC	NITE Biological Resource Center	Japan	ISO9001:2001
NCIMB	National Collection of Industrial, Food, Marine Bacteria	UK	ISO9001:2000
NCPV	National Collection of Pathogenic Viruses	UK	ISO 9001:2008
NCTC	National Collection of Type Cultures	UK	ISO 9001:2008
NCYC	National Collection of Yeast Cultures	UK	ISO 9001:2008
VTT	VTT Technical Research Centre of Finland Culture Collection	Finland	ISO 9001:2008

To ensure materials provided by the mBRC are authentic, processes must be under review to keep pace with taxonomy and advanced technology applications. These areas are under constant revision in order to increase the capacity to modify classification systems or identify new elements of biodiversity. mBRCs need investment in new technologies and human resources development to equip individuals with understanding, provide skills, access to information, knowledge and training that will enable them to perform their activities effectively. Consequently mBRCs must have sound operational financial plans to survive and to be independently sustainable. Part of the requirement of being a public service collection and of becoming an mBRC is that there is a back-up plan to ensure continuity of access to biological material used in research, deposited under confidential circumstances, or as part of publication of a patent. Thus, it is essential that the mBRCs have robust business plans for the long-term.

### Culture collection business plans

The financial challenge of developing Biological Resource Centres was recognized in the OECD’s report entitled *Biological Resource Centres–Underpinning the Future of Life Sciences and Biotechnology* (OECD 2001). This report made several recommendations encouraging co-ordination of activities among collections in response to the needs of the users of the microbial resources. It directed Governments to provide a baseline of long-term core funding to centres that qualify as BRCs to encourage high standards of quality and to promote research, development, new technology and commercial exploitation. To support the development of financial plans, the OECD suggested that various foundations and philanthropic or charitable organizations should be asked to extend the level of support given to BRCs. It was also considered appropriate to develop marketable products and services, including those aimed at meeting regulatory demands, and for sale to specialized customers, as long as they do not divert capacity from the core activities of BRCs. It was recognised that industry can play a part and should be persuaded to take a long-term view of their interests and to offer core support for BRCs, either through funding or through direct participation in the functioning of BRCs, provided the latter maintain their independence. Additionally, efforts to harmonize fee structures should be made in situations where fees are usually charged and to see that charges are affordable for users.

A workshop on Financial Aspects of BRC’s organised by the OECD Biological Resource Centres Task Force, Focus Group III in Genoa, 26 March 2003 identified typical income streams (Table 4). Some of these income streams are those that support existing models of culture collections whereas others represent activities in which most BRCs are still exploring and may participate in that may generate recoverable income from stakeholders.

**Table 4:** **Support for mBRCs**

Government support	Half the collections (over 300) listed by the World Data Centre for Microorganisms receive such support
Private industrial support for participation in the functioning of BRCs	Only 22 WDCM registered collections are supported by industry
Private industrial support for internal restricted BRC activities	Normally through bilateral contracts
Public and private foundation support	There are 40 collections that are privately supported
Public fundraising	Not many collections are exploiting this route
Sale of biological resources and technical materials	Most public service collections charge a supply fee; Often subsidised for the research community
Provision of specialist services and technical consulting expertise	330 collections deliver identification services and 284 provide consultancy
Research income (grants and contracts)	This varies from collection to collection and is dependent on the availability of researchers
Fees for repository service (safe deposits and patented strain maintenance)	100 collections receive patent deposits and 289 offer storage services
Provision of technical courses	283 WDCM registered collections offer training
Exploitation of and adding value to genetic resources	Rarely done by the collections
Provision of DNA, cDNA libraries, genomic libraries, filter sets, clones, plates, PCR products, RNAi resources	Only a small number of collections offer these as regular services
Data storage and retrieval, data mining tools	A specialist set of skills offered rarely

Data sourced 6 November 2013 from http://www.wfcc.info/ccinfo/statistics/.

However, it is debatable whether all these activities have a market and offer worthwhile returns. Often services are offered by specialist organisations and the market access can be very competitive.

The EMbaRC project examined sustainability of mBRCs comparing the revenue lines of the partner collections (Table 5). These collections are in the main well-established public service collections with a long history of providing products and services. They show that they have common products and services but the balance on how important each individual line is to each collection is significantly different.

**Table 5:** **EMbaRC partner collections**

Participating mBRCs	Country
Institut National de la Recherche Agronomique (INRA)	France
Institut Pasteur (IP)	France
Deutsche Sammlung von Mikroorganismen und Zellkulturen GmbH (DSMZ)	Germany
CAB International (CABI)	UK
Universitat de València (UVEG), Colección Española de Cultivos Tipo (CECT)	Spain
Universidade do Minho, Uminho-MUM	Portugal
Koninklijke Nederlandse Akademie Van Wetenschappen, Centraalbureau voor Schimmelcultures (CBS)	The Netherlands
BCCM Laboratorium voor Microbiologie (LMG), Universiteit Gent (UGent)	Belgium
BCCM Mycothèque de l’Université catholique de Louvain (MUCL)	Belgium

**Figure 1:**
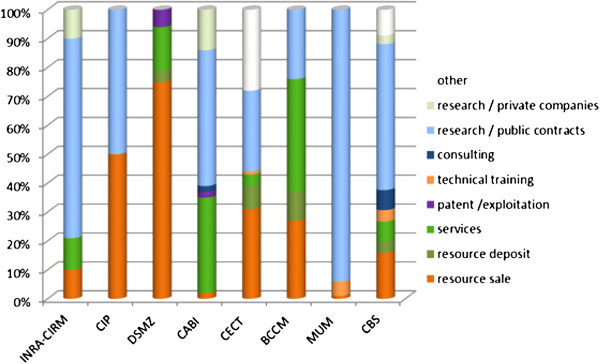
**Schematic display of funding sources for EMbaRC partner collections.**

The level of public funding was mainly in the range of 65-92% with the exception of CABI, which has no specific national funding for the collection; overall CABI has a member country contribution of around 3% (97% self-sufficient) and it invests in the collection maintenance activity. In general for the EMbaRC collections other sources of funding for 2009 were varied and are shown in Table 6 and Figure 1.

**Table 6:** **Microbial collections’ funding scheme**

Category of funding	Amount of funding
Maintenance & bench fees	0% to 16%
Resource supply	2% to 75%
Resource deposit	0% to 10%
Services	0% to 39%
Technical training	0% to 1%
Consulting	0% to 7%
Research/public contracts	0% to 94%
Research/private companies	0% to 14%

### Commercialisation

There is a requirement for adequate funding to support culture collections not only their continued maintenance but also their future development (OECD 2007). Traditional products must be complimented by the accession of new products to meet the needs of new users. Many collections are preparing DNA, enzymes, metabolites and other derivatives from authenticated strains, or curated databases linked to genome sequence either as standard inventory, or on a case-by-case basis. Collections can move beyond their historical context by developing commercial products through the provision of biotechnological solutions, active compounds and funding it through public/private investment and establishing spin off companies.

CABI has been moving in this direction since the 1990’s after direct UK Government funding ceased. For example, CABI identified the need for a rapid test kit to detect fungal contamination in kerosene because available detection methods of the time took as many as 3-10 days. The company Conidia Bioscience (http://www.conidia.com) was established to develop the FUELSTAT™ detection kit that is changing paradigms for detection of contaminants in fuel. The use of the FUELSTAT™ kit is recommended in the Boeing Aircraft Maintenance Manual demonstrating that it is not beyond culture collection staff to come up with solutions to current microbial problems and establish companies whose profits can be partly used to support biosystematics, biological collections and fundamental research.

CABI has also been involved in developing biocontrol agents and one particular success, in collaboration with partners, has been *Green Muscle* a product used for control of African Locust (Lomer et al. 2001). The profits from the sale of this product go into a fund to support biodiversity initiatives in Africa.

### Developing business plans

There are a number of ways mBRCs can develop their individual business plans. However, it is crucial that they do not become commercial entities; they must not compromise their public service role. With this caveat, some avenues that can be explored are outlined below. The collection must clearly define the range of products and services it can provide with the resources and expertise it has. These are outlined above as income streams. For example the mBRC might support governments to coordinate legitimate access to high quality resources for research and development and also to implement international conventions and legislation particularly in biosecurity and in regards to the Nagoya Protocol on Access and Benefit Sharing. Similarly, mBRCs may also support bio-industry, researchers and biotechnology by focussing essential services such as identification of novel organisms, targeting specific chemistry in organisms for further study and protection of public investment made in the isolation of organisms and the generation of information and knowledge by maintaining the link between the biological material and the information, and by being the honest broker in the conservation and utilisation of genetic resources.

The mBRC needs to understand the global and local market and seek information on whom and where their clientele are. There are over 650 collections listed in the World Data Centre for Microorganisms (WDCM) with over 2 million strains but there are gaps for example only 25.5% of types of the 100,000 fungal species described are available whereas there is 80% coverage of the types of 9,000 species of prokaryotes. Stackebrandt reported that of 20,200 prokaryotic research strains in 835 articles in eight European journals in 2008 only 190 strains (0.94%) were deposited in public service collections (Stackebrandt 2010). There is much work to do and focussing on the gaps can give an mBRC an advantage in the market. It is estimated that around half of a million strains are supplied each year by the collections registered in the WDCM. If 99% of strains supplied are not from collections then 50 million strains are exchanged often without provenance. While there is no quantitative estimate of the cost, in terms of misidentification, or the loss of indirect benefits, there is clearly a need for mBRCs to provide authenticated strains under a quality management system and with robust documentation of strain provenance. Moreover, there are also the yet to be described microbes to be considered. For example, there are 100,000 fungal species described but some estimates suggest that 1.4 million remain to be described and this gap is potentially larger for the Bacteria and Archaea. Innovative ways of addressing this will help mBRCs develop an appropriate and potentially successful business plan.

As much as there are opportunities for mBRCs to address, some of these come with associated risks and culture collections should balance the risk to established resources with the potential benefit of expanding into new areas. For example, in many areas there is pressure to consolidate resources into infrastructure level entities. While this has several benefits, usually expressed in terms of shared overhead, there is the opportunity for significant risk to impact established collections of materials. Chief among these risks is the loss of expertise associated with historical collections and this is usually expressed as an erosion in training of microbial taxonomists. While the number of species remaining to be characterized still vastly exceeds the number in collections, there will always be a need for microbial taxonomists. Similarly, while strains held in different collections may have shared historical provenance, their unique histories are important in regard to their status as reference materials, and even whether they are fit for use. While this may not have been evident in the pre-molecular era, molecular techniques are capable of differentiating between strains that have been otherwise considered equivalent. More recently, targeted gene sequencing (Wiest et al. 2012) and whole genome sequencing (McCluskey et al. 2011) of *Neurospora* has allowed unprecedented analysis of the relatedness of strains with shared lineage. Both of these examples reflect the research component, mandated in the OECD guidelines, but impossible in a large consolidated collection.

Other areas where risk needs to be managed include the investment in novel technologies, the impact of national and international legislation on exchange of materials (and information) and the false expectation that organisms will, in themselves, offer solutions to society’s challenges.

Some of these threats can be mitigated by engagement of local country authorities and some will depend on the broad expertise in the interested partners who will take up the opportunities and reduce impact of threats.

A sound financial plan is needed and funders need identifying and this is different in each country. Engagement at the Government level is not easy and a lot of work is needed to engage them. Not only do collections need to find novel ways of funding but also need to keep abreast and harness new technologies to produce information on the strains held, adding value with the aim to provide today’s users with the information they need. It is not always possible to establish these technologies in house but it is possible to establish partnerships with manufacturers, other collections or institutions with the expertise and facilities. Bioinformatics is of increasing importance to the operation of collections and new ways of collecting, storing, analysing, presenting and interrogating information are required to make best use of biodiversity information. Molecular techniques are increasing in use to differentiate between strains and in identification. However, work at CABI has shown through PCR fingerprinting with MR Primer of replicates of an isolate of *Metarhizium anisopliae* that after non-optimised preservation techniques were applied, polymorphisms were introduced (Ryan et al. 2002). Therefore at the very least collections should be adopting such techniques in their operations to determine if they are preserving strains without change.

Because most collections will have neither the means, nor the expertise to deliver materials and services across the range of microbial resources, networked mBRCs are the modern generation public service culture collections. The main drivers for the establishment of networks are the need to better utilise biological diversity in biotechnology to enable nations to deliver the Bioeconomy and deliver natural solutions to today’s global challenges. No single collection can address these challenges alone.

While a uniform structure of funding is not necessarily critical, many mBRCs will require a significant component of Government funding. Some guarantee of on-going funding is necessary to ensure that their essential functions remain reliable for R&D and support of biotechnology. It is essential that any networking activity does not put core mBRC activity at risk. mBRC networks can work towards the elimination of unnecessary duplication in holdings but their operational costs must not impact on the funding of individual member mBRCs. If the funding for individual collections is reduced the collection is rendered ineffective and can be closed and thus the network is weakened and undermined.

#### Strategic implications of operating a mBRC network

In order that a user gets consistent quality and reproducibility it is a prerequisite for any network that it is built upon common standards and accreditation. This defines the network and requires substantial investment. A national strategy that provides core financial support for a national mBRC (or mBRCs) reduces the need for excessive central costs. Managing a global network through a series of national hubs allows mBRCs to meet local requirements but also to take advantage of common approaches and outputs from the international mBRC network and provides a mechanism for the network to be sustainable. Being a network of networks ensures that the entire network doesn’t collapse if one component fails. The international mBRC network can be built upon national initiatives that in turn will evolve from existing activities. These activities are already based upon a range of income streams with varying levels of government support, these must be maintained.

Governments are fundamental partners in the creation of the national mBRCs and national mBRC networks that will contribute to the international network, regardless of the level of financial support needed. Not all culture collections will wish to neither become an mBRC nor participate in networks particularly if this is inappropriate to its aims or goals, or if this is not justifiable given the level of investment required to raise or alter standards. Links to enable the resources of such centres to be visible to the user community will need to be created with mechanisms to help them supply strains. Governments need to recognize that mBRCs will take a regulated role in the supply and maintenance of dangerous or pathogenic organisms. This important core aspect of mBRCs provides a controlled framework for the availability of these sensitive resources. In turn, fulfilling this role requires a level of financial commitment. mBRCs must use the opportunity of establishing an international network to seek sponsorship from a variety of new sources of support (national, international, public, private and industry).

### A proposed collaborative framework for mBRCs and long-term financial support

The German Government through Bundesministerium für Bildung und Forschung (BMBF), the German Federal Ministry of Research and Education supported a small Secretariat to draw national efforts together in developing tools for the establishment of a global network, the Global Biological Resource Centre Network (GBRCN). Since the report of these activities (Fritze et al. 2012), national and regional efforts have been initiated to begin the process of the establishment of the GBRCN and thus common policy to enhance microbiological research and improved mechanisms for uptake of quality microbial resources into biotechnology. The pan-European initiative, the Microbial Resources Research Infrastructure, furthers efforts and offers mechanisms for improvements in efficiency, sharing resources and expertise making partner mBRCs more cost effective (Smith 2012). To extend this to a global scale, it is envisaged that the GBRCN will be constructed by linking regional and national efforts. Efficiencies impacting on costs will be gained through a series of technical sub-groups or sub-committees to represent each area of technical expertise relevant to the operation of the mBRC network that would have roles in the standards, operation, quality control and data aspects of the network. These could include:

- Monitoring accreditation
- Advising organizations on standards
- Reviewing and recommending software packages for particular applications
- Providing a user interface with the mBRC network
- Providing a link to GBIF, the Global Biodiversity Information Facility

In order for the infrastructure to be sustainable, mBRCs need to create a robust management system. It is considered essential to do this at minimal cost and to avoid the unnecessary expense of establishing a large stand-alone secretariat. Once established it may be possible to have a more ‘virtual’ interaction and data access and exchange. An Interim Advisory Group may have to be set up prior to the nomination of the mBRC Management Board to facilitate the identification of participating organizations and call on expertise for this task drawn from a range of appropriate disciplines. This group would only exist for the period leading up to the launch of the project and have a defined remit, Terms of Reference and life span. MIRRI is taking up the challenge for Europe and is linked to the National Science Foundation funded US Culture Collection Network activities and keeping a watching brief on developments in Asia, South America and trying to establish activities with collaborators in Africa.

## Conclusion

mBRCs have to take a prominent role in capacity building and ensure a link between research-based collections, the mBRC, and the ultimate user. mBRC need to function as a strategic, national repository for key academic and industrial research resources, which will in turn provide an income stream. This is unlikely to operate on the basis of full cost recovery from sales income. A coordinated approach is necessary to ensure that a nation’s resources are secured for future use. Individual mBRCs will need to be flexible in their accession policies to work with others to take in the strains that are described in the scientific literature and are isolated or developed as a result of publicly funded research. In turn, Governments and funding agency policies must ensure that the products derived from their research programmes are deposited in mBRCs as part of the conditions attached to any award. Currently granting agencies are mandating data sharing and curation plans as part of any award. This same approach could ensure that microbial resources are preserved and made available. National funding agencies and mBRCs need to work together to provide greater support to research based collections in terms of training and advice on standards, quality control and integrate more with the national activities in key related priority research areas (e.g. model organism research consortia). This requires Governments to provide infrastructure funding. To ensure that the resources provided by the individual mBRC are fit for purpose and can be identified for specific use by biotechnologists, mBRCs must create partnerships with centres of excellence working with and developing new technologies to provide the necessary data. mBRC must also ensure that linkage is possible to data held in public databases so strain data can be mined alongside other information sources to facilitate innovation and discovery. It is also essential the mBRCs work with the data holders to ensure that the data is supported by physical resources held in mBRCs to facilitate validation of information or revisiting materials when new technologies become available.

It is anticipated that all of these strategic and operational changes relevant to the national role of mBRCs will enhance their position in providing services of benefit to the scientific community and thus in turn benefit them by maximizing the potential for financial support. A key element for discussion, however, remains the degree to which mBRCs may benefit from the direct commercial exploitation of the resources that they hold. ‘Ownership’ as a concept has, to a large degree, been avoided in the past with the mBRC acting as a ‘Custodian’ of the resource. Widespread introduction of Material Transfer Agreements and implications that IPR and reach-through are requirements for access to resources would fundamentally alter the relationship between Depositor, User and the mBRC.

Thus, there is not one financial model that can be applied to all culture collections, microbial domain BRC or mBRC. Existing structures suggest that a combination of governmental, commercial, and project portfolios offers the best chance for long-term sustainability. The investment is worth it, particularly if collections network for efficiency and work more closely with users to provide the resources needed.

## Abbreviations

BMBF: Bundesministerium für Bildung und Forschung (German Federal Ministry of Research and Education)
BRC: Biological Resource Centre
CABI: CAB International
CBS: Centraalbureau voor Schimmelcultures
cDNA: Complementary desoxyribonucleic acid
ESFRI: European Strategy Forum for Research Infrastructures
mBRC: Microbial Biological Resource Centres
MUCL: Mycothèque de l’Université Catholique de Louvain
OECD: Organisation for Economic Cooperation and Development
IMI: Imperial Mycological Institute
CMI: Commonwealth Mycological Institute
FGSC: Fungal Genetic Stock Center
WDCM: World Data Centre for Microorganisms
WFCC: World Federation for Culture Collections
EMbaRC: European Consortium for Microbial Resource Centres
MIRRI: The Microbial Resource Research Infrastructure
USCCN: United States Culture Collection Network
RCN: Research Coordination Network
MALDI-TOF: Matrix Assisted Laser Desorption/Ionization, Time of Flight
DSMZ: Deutsche Sammlung von Mikroorganismen und Zellkulturen GmbH (German Collection of Microorganisms and Cell Cultures)
IPR: Intellectual Property Rights
GBRCN: Global Biological Resource Centre Network
ISO: International Organization for Standardization.
